# A University-Led Community Program for Super-Aged Society

**DOI:** 10.1093/geroni/igaf122.752

**Published:** 2025-12-31

**Authors:** Noriko Yamamoto-Mitani, Yuka Sumikawa, Hanako Numata, Manami Takaoka, Taeko Mizukami, Yurina Miyano, Ayumi Igarashi

**Affiliations:** The University of Tokyo, Bunkyo, Tokyo, Japan; University of Tokyo, Bunkyo-City, Tokyo, Japan; University of Tokyo, University of Tokyo, Tokyo, Japan; The University of Tokyo, Bunkyo, Tokyo, Japan; Bunkyo City Social Welfare Council, Bunkyo City, Tokyo, Japan; Bunkyo City Social Welfare Council, Bunkyo City, Tokyo, Japan; The University of Tokyo, Tokyo, Tokyo, Japan

## Abstract

How to maintain a sustainable care delivery system for those who need care is an urgent issue for Japan, which has become a super-aged society and a society with a declining population. The solution to such social issues cannot be found through conventional research and surveys, but rather through community-based participatory research (CBPR), in which solutions are found together with citizens. Based on such a concept, the Global Nursing Research Center of the University of Tokyo School of Medicine is constructing a CBPR for community development aimed at extending life expectancy with well-being. This program will have: Care Competency Education Program, Room for Healthy Living, and Mutual Support for Care Professionals. In the Care Competency Education Program, nurses provide education on the ability to care for the health and safety of themselves and those around them. Room for Healthy Living will be a hub for health in the community, similar to the health centers in schools. It is a place where anyone can casually drop in and gather, and where they can easily consult with professional nurses as needed. It is a place where people with the same difficulties can get together and share information. Mutual Support for Care Professionals is a place where various professionals involved in care in the community can get together and see each other, deepen mutual understanding through case studies, etc., and learn the latest knowledge through lectures by researchers. The above initiatives are being implemented and developed based on the needs of citizens.

